# High-Throughput LC–MS/MS Quantification of Eighteen Cannabinoids in Hemp Flowers with Baseline Separation of Structural Isomers

**DOI:** 10.3390/molecules31101684

**Published:** 2026-05-16

**Authors:** Na Liu, Maggie Schoener, Naima Jannath Rimi, Md Imon Hossain, Supraja Regunathan, Robert Powers, Liguo Song

**Affiliations:** 1 Department of Physical and Biological Sciences, Western New England University, Springfield, MA 01119, USA; 2 Department of Chemistry, Western Illinois University, Macomb, IL 61455, USA; me-schoener@wiu.edu (M.S.); n-rimi@wiu.edu (N.J.R.); i-hossain3@wiu.edu (M.I.H.); 3 Department of Forensic Science, University of New Haven, West Haven, CT 06516, USA; suprajaregunathan@gmail.com (S.R.); rpowers@newhaven.edu (R.P.)

**Keywords:** liquid chromatography, triple-quadrupole mass spectrometer, cannabinoids, quantification, hemp flowers, MRM

## Abstract

Following the passage of the Agriculture Improvement Act of 2018, demand for accurate cannabinoid quantification in hemp flowers has increased to ensure regulatory compliance. Liquid chromatography–tandem mass spectrometry (LC–MS/MS) using a triple-quadrupole mass spectrometer provides high sensitivity and selectivity and is well suited for this purpose; however, a review of the literature indicates that many published LC–MS/MS methods target only a limited number of cannabinoids, and reliable differentiation of structural isomers remains challenging. In this study, an LC–MS/MS method was developed for the simultaneous quantification of eighteen cannabinoids in hemp flowers. Baseline chromatographic separation of structural isomers enabled reliable differentiation of compounds with highly similar fragmentation patterns and allowed the use of the most sensitive multiple reaction monitoring (MRM) transitions for quantification. Both positive and negative ionization modes were employed to achieve optimal sensitivity using dynamic polarity switching within a single analytical run. Following validation in accordance with ISO/IEC 17025, the method was applied to a proficiency test hemp sample and six commercial hemp samples, demonstrating excellent time efficiency (11 min for 18 cannabinoids) and an exceptionally wide calibration range (8–5000 ng/mL, corresponding to 0.032–20% (*w*/*w*) for all cannabinoids).

## 1. Introduction

*Cannabis sativa* L. is a flowering plant species in the family *Cannabaceae*, commonly referred to as cannabis [1]. Some cannabinoids also have important medical applications [2]; for example, cannabidiol (CBD) is approved for the treatment of certain forms of epilepsy [3]. Other constituents include terpenes, which contribute to aroma [2,4], and non-cannabinoid phenols and alkaloids.

Cannabinoids are primarily synthesized and accumulated in glandular trichomes on female flowers, where they reach their highest concentrations [5]. Under the Agriculture Improvement Act of 2018, hemp is defined in the United States as *Cannabis sativa* L. containing no more than 0.3% (*w*/*w*) Δ^9^-THC on a dry weight basis. Because cannabinoids are concentrated in female flowers, hemp flower is a major commercial product subject to routine compliance testing. The 0.3% (*w*/*w*) Δ^9^-THC limit is defined as the sum of Δ^9^-THC and its acidic precursor, Δ^9^-tetrahydrocannabinolic acid (Δ^9^-THCA), expressed as Δ^9^-THC equivalents.

Although Δ^9^-THC and Δ^9^-THCA are the primary analytes for regulatory purposes, other cannabinoids, including CBD, cannabigerol (CBG), cannabichromene (CBC), cannabinol (CBN), cannabicyclol (CBL), cannabidivarin (CBDV), and tetrahydrocannabivarin (THCV)—as well as their corresponding acidic precursors, including cannabidiolic acid (CBDA), cannabigerolic acid (CBGA), cannabichromenic acid (CBCA), cannabinolic acid (CBNA), cannabicyclolic acid (CBLA), cannabidivarinic acid (CBDVA), and tetrahydrocannabivarinic acid (THCVA), are also relevant. Additional minor cannabinoids, such as Δ^8^-THC and cannabitriol (CBT), may also be present in hemp flower. The presence of these structurally diverse cannabinoids, many of which are reported for labeling or commercial purposes, necessitates comprehensive and accurate analytical methods for cannabinoid quantification in hemp flowers to ensure regulatory compliance and quality control [6].

Gas chromatography coupled with flame ionization detection (GC-FID) or mass spectrometry (GC-MS) has been used historically for cannabinoid analysis [7,8,9,10,11]. However, these methods have limitations because thermal decarboxylation of acidic cannabinoids occurs during analysis, which is often incomplete and variable, leading to partial conversion of acidic cannabinoids into their neutral forms and compromising accurate quantification; therefore, derivatization is required for reliable analysis of acidic cannabinoids.

Cannabinoid analysis has also been performed using liquid chromatography with ultraviolet or diode array detection (LC-UV/DAD) [12,13,14,15,16,17]. Although these methods are widely used due to their cost-effective instrumentation, they do not provide definitive confirmation of analyte identity. In addition, their limited selectivity necessitates baseline separation of all analytes present above the limit of quantification (LOQ) to avoid quantification interferences, which limits analytical throughput. Although acceptable selectivity is often reported, minor interferences still occur.

Liquid chromatography–high-resolution mass spectrometry (LC-HRMS), including quadrupole time-of-flight (QTOF) and quadrupole-Orbitrap instruments, has also been used for the quantification of cannabinoids in a variety of matrices, such as edibles, beverages, and oils [18,19,20]. However, dynamic polarity switching is generally avoided in HRMS-based quantitative methods, as it is constrained by duty cycle and scan speed, which can adversely affect sensitivity and quantitative accuracy in multi-analyte analyses.

LC–MS/MS with triple-quadrupole instruments has also been used for the quantification of cannabinoids [21,22,23]. In some reports, coelution of Δ^8^-THC and Δ^9^-THC was observed, and attempts were made to differentiate and quantify these isomers using distinct MRM transitions [23]. However, subsequent studies have indicated that this approach may not provide reliable differentiation, as the two isomers exhibit nearly identical fragmentation patterns. To the best of our knowledge, the maximum number of cannabinoids quantified in a single run using LC–MS/MS with a triple-quadrupole mass spectrometer is seventeen, with a separation time of 16 min [21].

**Figure 1:**
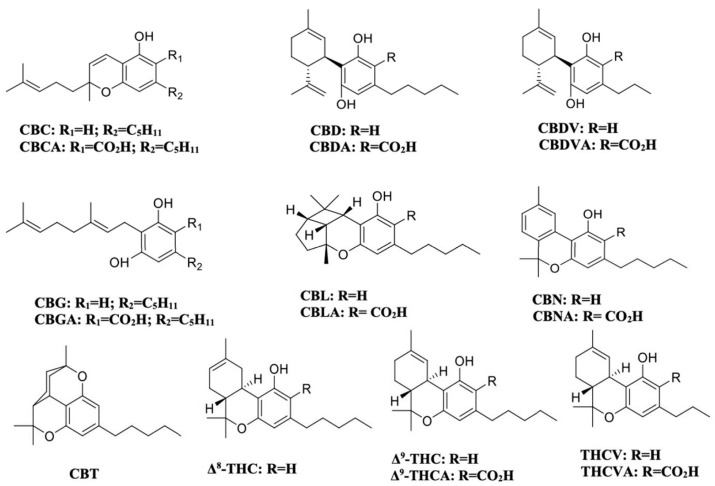
Chemical structures of the eighteen cannabinoids investigated in this study.

Given the limitations of existing analytical approaches, this study aims to develop a high-throughput method for the quantification of eighteen cannabinoids (Figure 1) in hemp flowers, representing, to the best of our knowledge, the largest panel reported for a single analytical run. The additional analyte included in this work is CBT, which has been increasingly detected in hemp flower samples [24,25]. The inclusion of CBT presents an added chromatographic challenge due to the presence of multiple structurally related isomers (e.g., CBD, Δ^9^-THC, Δ^8^-THC, CBL, and CBC). Achieving baseline separation of these compounds while maintaining a short analysis time requires careful optimization of chromatographic conditions.

LC–MS/MS with a triple-quadrupole mass spectrometer was selected due to its ability to provide definitive confirmation of analyte identity, enable dynamic polarity switching within a single run, and selectively differentiate and quantify cannabinoids using MRM transitions. The use of isotopically labeled internal standards (Supplementary Figure S1) further minimizes matrix effects and supports a wide calibration range. Additionally, the method developed in this work achieves baseline chromatographic separation of structural isomers, enabling their reliable differentiation and accurate quantification in complex hemp flower matrices.

## 2. Results and Discussion

### 2.1. Method Development

All eighteen targeted cannabinoids and nine commercially available isotopically labeled cannabinoid internal standards from MilliporeSigma (Burlington, MA, USA) were individually optimized to determine the optimal MS/MS parameters for each analyte. The isotopically labeled internal standards were selected based on their structural similarity and comparable chromatographic and ionization behaviors to the target cannabinoids. Neutral cannabinoids exhibited better performance in positive ionization mode; however, several analytes, particularly acidic cannabinoids, showed improved response in negative mode, with significantly higher signal intensity than in positive mode. Given the instrument’s ability to efficiently switch between positive and negative ionization modes, both modes were utilized in the method. Neutral cannabinoids were analyzed in positive mode, while most acidic cannabinoids were analyzed in negative mode. Optimal MS/MS parameters for individual cannabinoids and isotopically labeled internal standards (I.S.), including ionization mode (positive or negative), retention time (RT), precursor ions, quantifier ions, qualifier ions, and collision energies (CEs) for each transition, are summarized in Table 1**,** respectively.

**Figure 2:**
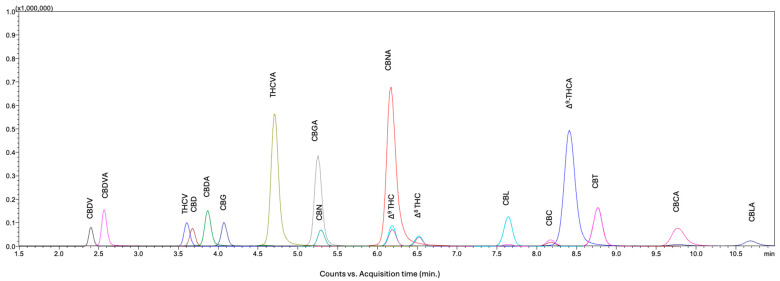
A representative LC–MS/MS chromatogram of eighteen cannabinoids at an individual concentration of 100 ng/mL.

**Figure 3:**
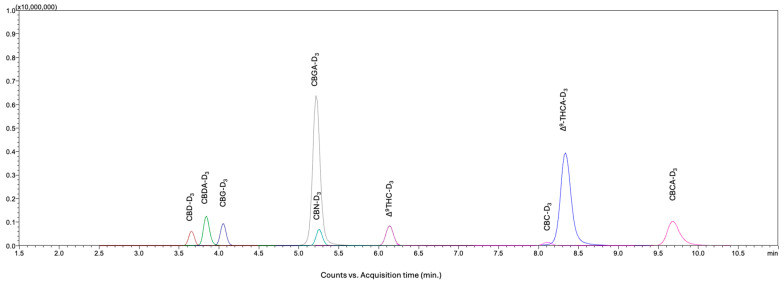
A representative LC–MS/MS chromatogram of the nine isotopically labeled internal standards at the individual concentration of 500 ng/mL.

Following individual MS/MS optimization for each cannabinoid and I.S., emphasis was placed on achieving optimal chromatographic separation. Owing to the similar optimized MS/MS transition parameters observed for structural isomers, particularly the Δ^9^-THC and Δ^8^-THC stereoisomer pair, additional efforts were directed toward attaining their baseline separation. Based on the previously achieved separation [26], the mobile phase consisted of solvent A (0.01% formic acid and 1 mM ammonium formate in water) and solvent B (acetonitrile). Various proportions of solvent B (68–71% *v*/*v*) were systematically evaluated, and 70% (*v*/*v*) solvent B provided baseline resolution of all structural isomers, including Δ^9^-THC and Δ^8^-THC, within the shortest run time (11 min). A representative LC–MS/MS chromatogram of the eighteen cannabinoids at an individual concentration of 100 ng/mL is presented in Figure 2. A representative LC–MS/MS chromatogram of the nine isotopically labeled I.S. at the individual concentration of 500 ng/mL is presented in Figure 3.

To further support the assessment of chromatographic separation, LC–MS/MS chromatograms for cannabinoids sharing identical precursor ions are provided in Supplementary Figures S2–S5, with resolution (Rs) values calculated between adjacent peaks within each group. These groups include neutral C21 cannabinoids (*m*/*z* 315.20; CBD, Δ^9^-THC, Δ^8^-THC, CBC, CBL, and CBT), acidic C21 cannabinoids (*m*/*z* 359.20 and 357.20 for positive and negative mode, respectively; CBDA, Δ^9^-THCA, CBCA, and CBLA), neutral varin cannabinoids (*m*/*z* 287.20; CBDV and THCV), and acidic varin cannabinoids (*m*/*z* 331.20 and 329.20 for positive and negative mode, respectively; CBDVA and THCVA). Baseline separation was achieved for cannabinoids within these groups, with the smallest Rs value of 1.89 observed for the Δ^9^-THC/Δ^8^-THC pair, which are not readily distinguishable by MS/MS alone due to shared precursor ions and similar fragmentation behavior. For the remaining cannabinoids (CBG, CBGA, CBN, and CBNA; *m*/*z* 317.20, 359.20, 311.20, and 353.00, respectively), coelution with analytes in the defined precursor-ion groups does not affect accurate identification or quantification, as these compounds are distinguished by their precursor and MRM transitions.

Peak purity was evaluated by monitoring MRM transitions for each analyte. The qualifier-to-quantifier ion ratios were consistent across the chromatographic peak, and all transitions co-eluted at identical retention times with consistent peak shapes, indicating the absence of detectable co-eluting interferences. This approach provides both chromatographic and mass spectrometric selectivity in LC–MS/MS, where analyte identification and confirmation are achieved through combined retention time matching, multiple MRM transitions, and ion ratio consistency.

### 2.2. Method Validation

Calibration curves were constructed over the range of 8–5000 ng/mL using 1/x^2^ weighted linear regression to account for heteroscedasticity across the concentration range. The use of a single, wide calibration range is intentional and aligns with previous reports identifying the use of two calibrations as a limitation in cannabinoid analysis [27]. In this study, a single calibration curve was employed to enable simultaneous quantification of cannabinoids present at substantially different concentrations, thereby improving analytical efficiency and consistency across analytes. Acceptable linearity was achieved for all analytes, with coefficients of determination (R^2^) ranging from 0.9831 to 0.9996 (Supplementary Table S1). Calibration performance was further evaluated using back-calculated concentrations at each level, all of which met predefined acceptance criteria (±15% of nominal values and ±20% at the lowest calibration level). Method accuracy and precision were evaluated using quality control (QC) samples at 8, 200, and 5000 ng/mL. QC samples were analyzed in triplicate across three separate days. The resulting accuracy and precision data are summarized in Supplementary Tables S2 and S3, respectively. All results met the acceptance criteria defined by ISO/IEC 17025 [28].

Accuracy was expressed as percent of nominal concentrations, calculated from triplicate measurements for both intraday and interday analyses. The highest and lowest accuracy values at each QC level were identified and highlighted in red in Supplementary Table S2. At the 8 ng/mL QC level, intraday accuracy ranged from 86.4% to 116.8%, while interday accuracy ranged from 92.9% to 111.4%. At the 200 ng/mL QC level, the intraday accuracy range was slightly narrower than that at 8 ng/mL, ranging from 86.3% to 113.1%, while interday accuracy ranged from 90.0% to 112.0%. At the highest QC level (5000 ng/mL), intraday accuracy ranged from 85.6% to 114.3%, and interday accuracy ranged from 86.6% to 113.3%.

Precision was evaluated by Relative Standard Deviation (RSD) values which were computed using triplicate measurements for both intraday and interday. The highest RSD at each QC level was identified and highlighted in red in Supplementary Table S3. At 8 ng/mL QC level, highest RSD for intraday and interday were 10.7% and 9.3% respectively; while at 200 ng/mL QC level, RSDs were much tighter and highest RSD for intraday and interday were 5.1% and 6.8% respectively; at highest QC level (5000 ng/mL), highest RSD for intraday was 5.9% which was similar to the middle QC level, while highest RSD for interday was 10.2%.

Supplementary Table S4 summarizes uncertainty estimates for cannabinoid quantification based on replicate measurements of QC samples (Supplementary Table S3). Expanded uncertainty values were calculated using a coverage factor (k = 2), corresponding to an approximate 95% confidence interval, in accordance with ISO/IEC 17025 guidelines.

The signal-to-noise (S/N) ratios of all cannabinoids at the lowest calibration level (8 ng/mL) were evaluated, and the limits of detection (LOD) and quantification (LOQ) were determined based on S/N criteria of 3:1 and 10:1, respectively. The results are summarized in Table 2, together with the calibration curve equations and corresponding linear ranges. The LOD and LOQ obtained for cannabinoids analyzed in positive ionization mode are comparable to those reported in previously published LC–MS/MS methods [29,30,31]. For selected acidic cannabinoids, the use of negative ionization mode, implemented via dynamic polarity switching within a single run on a triple-quadrupole mass spectrometer, resulted in improved sensitivity, as reflected by lower LOD and LOQ values [29].

### 2.3. Analysis of Hemp Proficiency Testing (PT) Flower Samples

To further evaluate method accuracy and performance on a real-world matrix, a hemp PT flower sample from the Hemp Proficiency Testing Program at the University of Kentucky was analyzed. The sample was processed in triplicate according to the procedure described in the Materials and Methods section, yielding three independent extracts. The extraction protocol has been extensively validated and applied in prior studies [12,18,32,33,34], demonstrating high extraction recoveries with satisfactory intra- and inter-day precision, thereby supporting its suitability and reliability for comprehensive cannabinoid analysis.

**Figure 4:**
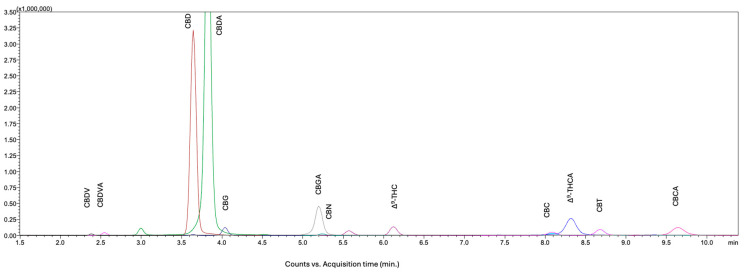
A representative LC-MS/MS chromatogram of the hemp PT flower extract at concentration of 25,000 ng/mL.

The LC-MS/MS chromatogram of the sample is shown in Figure 4. The quantitative analytical results and their comparison with other labs are summarized in Table 3.

Although a large number of laboratories participated in the hemp PT study, most employed LC–UV/DAD methods. Only four laboratories used LC–MS methods: two reported CBD, CBDA, Δ^9^-THC, Δ^9^-THCA and CBN; one reported CBD, CBDA, Δ^9^-THC, and Δ^9^-THCA; and one reported Δ^9^-THC and Δ^9^-THCA. All results were based on triplicate analyses.

In Table 3, the mean (w%) and RSD% for each laboratory and for the interlaboratory dataset are summarized alongside the corresponding values obtained in this study. Method bias was calculated by comparing the mean values obtained in this study with the corresponding interlaboratory mean values. The rows are arranged in descending order of interlaboratory mean cannabinoid concentrations. Total CBD and total Δ^9^-THC are reported at the end of the table and were calculated as the sum of the neutral cannabinoids and their decarboxylated acidic precursors (e.g., total CBD = CBD + 0.877 × CBDA).

Several observations can be made from the data presented in Table 3. The method employed in this study demonstrated good analytical performance, including good repeatability (RSD typically < 10%) and the use of isotopically labeled internal standards to closely match analyte behavior. Chromatographic conditions were optimized to achieve baseline separation of structural isomers, and calibration solutions were prepared using pre-mixed cannabinoid standards. Collectively, these features help minimize common sources of analytical variability, including matrix effects, ionization variability, and coelution, consistent with previous findings demonstrating that isotope dilution effectively compensates for matrix-induced ionization variability [18].

The interlaboratory data show differing levels of variability across analytes. Relatively low variability was observed for Δ^9^-THC, Δ^9^-THCA, total Δ^9^-THC, and CBN (RSD < 15%), suggesting consistent agreement among participating laboratories. CBD showed similar variability (RSD = 19%). In contrast, CBDA exhibited substantially higher variability (RSD > 30%), which is also reflected in the higher variability observed for total CBD (RSD = 24.7%).

In comparison with interlaboratory mean values, the results obtained in this study showed good agreement for analytes such as CBN, CBD, and total CBD. CBDA exhibited a moderate negative bias (−21.2%); however, given its higher interlaboratory variability, this deviation falls within the broader dispersion observed across laboratories. In contrast, a consistent negative bias was observed for Δ^9^-THC (−23.2%), Δ^9^-THCA (−41.9%), and total Δ^9^-THC (−27.0%).

These differences may reflect variability in analytical approaches across laboratories, including differences in selectivity, analyte coverage, and analytical procedures. However, given the limited number of laboratories and differences in analytes reported across laboratories, and in the absence of established reference values, the source of the observed discrepancies—including the possibility of method-specific bias—cannot be definitively established.

Overall, these results highlight challenges in interlaboratory comparability in cannabinoid analysis and indicate that further work is needed to improve method harmonization and standardization.

### 2.4. Potency Analysis of Six Commercial Hemp Flower Samples

The validated method was further applied to the potency analysis of six commercial hemp flower samples: Chem Berry Smalls (CBS), Classic Cookies Smalls (CCS), Forbidden V Smalls (FVS), La Crema Smalls (LCS), Strawberry Jam Smalls (SJS), and White CBG Smalls (WCS). These samples were selected based on their marketed cannabinoid profiles, including CBD-rich (CBS), CBDV-rich (CCS, FVS, and SJS), and CBG-rich (LCS and WCS) hemp flowers, as described by the vendor. Subsequent analysis was conducted to quantitatively evaluate their cannabinoid composition. The samples were extracted following the same procedure used for the hemp PT flower and analyzed using the validated method. All samples were prepared in triplicate, and the measured means and RSDs for cannabinoids are summarized in Table 4. Representative LC–MS/MS chromatograms of each hemp flower extract at 25,000 ng/mL are shown in Supplementary Figures S6–S11.

All major constituents present at levels greater than 1% (*w*/*w*) are highlighted in red in Table 4. CBDA was identified as the predominant constituent in four of the six samples (CBS, CCS, FVS, and SJS), whereas CBGA was the principal component in the remaining two samples (LCS and WCS). Among the CBDA-dominant flowers, three samples (CCS, FVS, and SJS) contained notable levels of CBDVA (1.5%, 5.3%, and 2.8%, respectively), consistent with their marketed CBDV-rich designation, although CBDA remained the dominant cannabinoid in each case. The CBG-dominant profiles observed for LCS and WCS are consistent with the accumulation of the biosynthetic precursor CBGA due to limited downstream conversion. In contrast, the lower abundance of CBDV-type cannabinoids relative to CBD is consistent with the known biosynthetic preference for C_5_ precursor pathways (olivetolic acid-derived), whereas CBDV-type cannabinoids originate from the less favored C_3_ precursor pathway (divarinolic acid-derived).

All samples contained less than 0.3% Δ^9^-THC, consistent with vendor-designated compliance. However, this designation appears to be based on Δ^9^-THC alone rather than total Δ^9^-THC, as all samples also contained measurable levels of Δ^9^-THCA that contribute to total Δ^9^-THC upon decarboxylation. Consequently, three samples (CBS, CCS, and SJS) exhibited total Δ^9^-THC contents exceeding 0.3% when calculated on a decarboxylated basis. Notably, the relative order of total Δ^9^-THC across samples paralleled that of total CBD, reflecting their shared origin from the CBGA biosynthetic pathway [35,36,37]. This trend is consistent with differential partitioning of CBGA into downstream cannabinoids, where increased conversion toward CBDA is associated with higher Δ^9^-THCA levels, while diversion toward CBDV-type cannabinoids or retention as CBGA corresponds to lower Δ^9^-THCA levels [35,36,37]. These observations are consistent with known biosynthetic relationships and with trends reported in our previous studies [24,25]. These results highlight the importance of accounting for acidic precursors in regulatory assessments. Accordingly, these observations are presented as compositional trends rather than mechanistic conclusions.

## 3. Materials and Methods

### 3.1. Chemicals, Standards and Hemp Flowers

LC-grade solvents (water, methanol, and acetonitrile) were purchased from Fisher Scientific (Waltham, MA, USA). Mobile phase additives, including LC-grade formic acid and ammonium formate, were purchased from MilliporeSigma (Burlington, MA, USA) and Fisher Scientific, respectively.

All certified reference materials (CRMs) were manufactured by Cerilliant and purchased from MilliporeSigma, including a mixture containing 17 cannabinoids, each at 250 µg/mL (0.1% ascorbic acid and 1% DIPEA in 50:50 MeOH:ACN), comprising CBC, CBCA, CBL, CBLA, CBD, CBDVA, CBG, CBN, CBNA, CBDA, CBDV, CBGA, (−)-Δ^8^-THC, (−)-Δ^9^-THC, THCVA, Δ^9^-THCA, and THCV, as well as an individual CRM of CBT (1 mg/mL in acetonitrile). All isotopically labeled cannabinoid internal standards (100 µg/mL) were also manufactured by Cerilliant and purchased from MilliporeSigma, including CBC-D_3_ (in methanol), CBCA-D_3_ (1% DIPEA and 0.05% ascorbic acid in acetonitrile), CBD-D_3_ (in methanol), CBDA-D_3_ (1% DIPEA and 0.05% ascorbic acid in acetonitrile), CBG-D_3_ (in methanol), CBN-D_3_ (in methanol), CBGA-D_3_ (1% DIPEA and 0.05% ascorbic acid in acetonitrile), Δ^9^-THC-D_3_ (in methanol), and Δ^9^-THCA-D_3_ (in acetonitrile).

Hemp PT Sample HM24NOV-1 was purchased from the Hemp Proficiency Testing Program at the University of Kentucky (Lexington, KY, USA).

Six hemp flower samples, including CBS, CCS, FVS, LCS, SJS, and WCS, were purchased from Tweedle Farms (Jacksonville, OR, USA).

### 3.2. Standard Solution Preparation

An isotopically labeled internal standard mixture was prepared in methanol to contain the nine isotopically labeled cannabinoids described above, each at a concentration of 1000 ng/mL. Nine calibration standard mixtures of the eighteen cannabinoids were prepared in methanol at 16, 40, 80, 200, 400, 1000, 2000, 5000, and 10,000 ng/mL for each cannabinoid and subsequently mixed 1:1 with the internal standard solution to yield final concentrations of 8, 20, 40, 100, 200, 500, 1000, 2500, and 5000 ng/mL, with each internal standard at a final concentration of 500 ng/mL. In-house QC samples were prepared in the same manner at twice the target concentrations (16, 400, and 10,000 ng/mL) for each cannabinoid to obtain final concentrations of 8, 200, and 5000 ng/mL.

### 3.3. Hemp Flower Sample Preparation

Both the hemp PT sample and hemp-based flower samples were prepared following the procedure described below. Approximately 7 g (¼ oz) of flower sample was initially ground for 2 min using a Waring laboratory blender (Torrington, CT, USA). Approximately 0.5 g of the ground material was then further pulverized by shaking at 3000 rpm for 2 min using a SPEX GenoLyte 1200 (Metuchen, NJ, USA) in a 7 mL tube containing two ¼-inch stainless steel balls. For cannabinoid extraction, approximately 100 mg of the powdered sample was weighed into a 15 mL centrifuge tube and suspended in methanol to obtain a concentration of 25 mg/mL. The suspension was ultrasonicated for 5 min and briefly vortexed to rinse any material from the tube walls. This ultrasonication–vortexing cycle was repeated four times. Approximately 2 mL of the resulting supernatant was then centrifuged at 13,000 rpm for 10 min, followed by filtration of ~1 mL of the supernatant through a 0.2 µm polytetrafluoroethylene (PTFE) syringe filter (Foxx Life Sciences, Salem, NH, USA). The filtrate was serially diluted with methanol (10×, 10×, and 5×), yielding a final sample solution equivalent to 50,000 ng/mL of original sample. An aliquot of 100 µL of this solution was mixed with 100 µL of an isotopically labeled cannabinoid internal standard mixture (1000 ng/mL of each of the nine analytes in methanol), producing a final solution containing 25,000 ng/mL of sample. The mixed solution was analyzed by LC-ESI-MS/MS. Cannabinoid concentrations were determined using calibration curves ranging from 8 to 5000 ng/mL for individual cannabinoids. The linear range for individual cannabinoids in the sample corresponded to 0.032–20% (*w*/*w*).

### 3.4. LC-MS/MS Instrumentation

Analysis was performed using a Shimadzu LCMS-8050 system (Columbia, MD, USA) at the University of New Haven equipped with LC-20ADXR binary pumps, solvent degassers, mixing vessels, an autosampler (SIL-20ACXR), a column oven (CTO-20AC), an ESI source, and a triple-quadrupole tandem mass spectrometer (MS/MS).

Chromatographic separation was achieved using a Waters CORTECS Shield RP18 column (90 Å, 2.7 µm, 2.1 × 150 mm; Milford, MA, USA), protected by a Restek UltraShield UHPLC precolumn filter (0.2 µm frit; Bellefonte, PA, USA). The column temperature was maintained at 30 °C. An isocratic mobile phase consisting of 30% (*v*/*v*) solvent A (0.01% formic acid and 1 mM ammonium formate in water) and 70% (*v*/*v*) solvent B (acetonitrile) was used at a flow rate of 0.3 mL/min. The total run time was 11 min, and the injection volume was 2 µL.

The ESI source parameters were as follows: interface temperature, 300 °C; nebulizing gas flow, 3.0 L/min; heating gas flow, 10.0 L/min; heat block temperature, 400 °C; desolvation line temperature, 250 °C; drying gas flow, 10.0 L/min.

Method validation was performed by evaluating linearity, accuracy, and precision using calibration standards and QC samples. Calibration curves were constructed over the range of 8–5000 ng/mL. Accuracy and precision were assessed at three QC levels (8, 200, and 5000 ng/mL) using triplicate measurements across three separate days. Accuracy was expressed as percent of nominal concentration, and precision as relative standard deviation (RSD). Acceptance criteria were defined as R^2^ ≥ 0.98, accuracy within 85–115%, and RSD ≤ 15%, with wider limits of 80–120% for accuracy and RSD ≤ 20% applied at the lowest QC level, consistent with ISO/IEC 17025 principles.

## 4. Conclusions

A high-throughput LC–MS/MS method was developed for the simultaneous quantification of eighteen cannabinoids in hemp flower matrices, incorporating baseline chromatographic separation of structural isomers and the use of all commercially available isotopically labeled internal standards from MilliporeSigma. The method demonstrated good precision (RSD < 10%) and enabled reliable differentiation of structural isomers within an 11 min analysis.

Application to a hemp proficiency testing sample showed generally good agreement with interlaboratory LC–MS results for CBD, CBN, and total CBD, while differences were observed for Δ^9^-THC (−23.2%), Δ^9^-THCA (−41.9%), and total Δ^9^-THC (−27.0%). CBDA exhibited a −21.2% deviation; however, this occurred in the context of substantially higher interlaboratory variability (RSD 33.4%). These results underscore the need for further interlaboratory evaluation using well-characterized reference materials and highlight the importance of transparent reporting of method parameters to improve method comparability.

Analysis of six commercial hemp flower samples revealed cannabinoid profiles consistent with biosynthetic pathway partitioning, which influences total Δ^9^-THC outcomes. Although all six commercial hemp flower samples contained Δ^9^-THC below 0.3%, three exceeded this threshold when expressed as total Δ^9^-THC, suggesting that acidic precursors are not consistently considered in vendor compliance determinations and highlighting the need for clearer regulatory guidance. The results further indicate that the likelihood of exceeding total Δ^9^-THC thresholds is influenced by cannabinoid composition: CBD-dominant profiles exhibit the highest total Δ^9^-THC, whereas increased CBDV content is associated with lower total CBD and reduced total Δ^9^-THC, and CBGA-dominant profiles exhibit the lowest total Δ^9^-THC.

## Figures and Tables

**Table 1:** Optimized MS/MS acquisition and quantification parameters for targeted cannabinoids and I.S. The dwell time was 20 ms.

Analytes	Mode	RT	Precursor *m*/*z*	Quantifier *m*/*z*	Quantifier CE (ev)	Qualifier *m*/*z*	Quallifier CE (ev)	I.S.
CBDV	+	2.40	287.20	165.10	25.0	231.10	20.0	CBD-D3
CBDVA	+	2.57	331.20	313.20	20.0	191.10	30.0	CBD-D3
THCV	+	3.61	287.20	165.10	25.0	231.10	20.0	CBD-D3
CBD	+	3.67	315.20	193.10	25.0	259.20	20.0	CBD-D3
CBD-D3	+	3.65	318.20	196.10	20.0	262.20	11.0	N/A *
CBDA	+	3.87	359.20	341.20	20.0	219.10	30.0	CBDA-D3
CBDA-D3	+	3.84	362.20	344.20	13.0	222.10	13.0	N/A
CBG	+	4.07	317.20	193.10	40.0	123.00	35.0	CBG-D3
CBG-D3	+	4.06	320.30	196.10	20.0	123.05	35.0	N/A
THCVA	−	4.70	329.20	285.10	20.0	217.10	25.0	CBGA-D3
CBGA	−	5.25	359.20	315.20	22.0	297.20	25.0	CBGA-D3
CBGA-D3	−	5.21	362.20	344.20	21.0	318.25	22.0	N/A
CBN	+	5.28	311.20	223.10	25.0	293.20	20.0	CBN-D3
CBN-D3	+	5.24	314.20	223.10	24.0	296.20	19.0	N/A
Δ^9^-THC	+	6.17	315.20	193.10	25.0	259.20	20.0	Δ^9^-THC-D3
Δ^9^-THC-D3	+	6.14	318.20	196.10	25.0	135.10	21.0	N/A
CBNA	−	6.18	353.00	279.20	35.0	309.15	35.0	CBGA-D3
Δ^8^-THC	+	6.52	315.20	193.10	25.0	259.20	20.0	Δ^9^-THC-D3
CBL	+	7.64	315.20	235.20	20.0	81.10	30.0	CBDA-D3
CBC	+	8.17	315.20	193.10	20.0	123.10	34.0	CBC-D3
CBC-D3	+	8.11	318.20	196.25	24.0	123.10	34.0	N/A
Δ^9^-THCA	−	8.41	357.20	313.15	25.0	245.20	35.0	Δ^9^-THCA-D3
Δ^9^-THCA-D3	−	8.34	360.20	316.25	25.0	248.15	30.0	N/A
CBT	+	8.77	315.20	193.10	25.0	259.20	20.0	CBDA-D3
CBCA	−	9.77	357.20	191.20	35.0	203.10	35.0	CBCA-D3
CBCA-D3	−	9.68	360.20	342.05	21.0	194.20	28.0	N/A
CBLA	+	10.68	359.20	341.20	20.0	261.10	25.0	Δ^9^-THC-D3

* N/A, not applicable.

**Table 2:** Limit of quantification (LOQ), limit of detection (LOD), calibration curve equation and correlation coefficient (R^2^) obtained from an analytical method validation procedure. All analytes exhibited a linear calibration range of 8–5000 ng/mL.

Analyte	LOQ (ng/mL)	LOD (ng/mL)	Calibration Curve	R^2^
CBDV	0.54	0.16	y = 0.001535x + 0.004960	0.9983
CBDVA	0.67	0.20	y = 0.002043x + 0.004037	0.9929
THCV	0.36	0.11	y = 0.003428x + 0.004805	0.9937
CBD	0.57	0.17	y = 0.002301x + 0.001701	0.9912
CBDA	0.54	0.16	y = 0.002122x + 0.005156	0.9945
CBG	0.65	0.19	y = 0.001755x + 0.004956	0.9996
THCVA	0.08	0.03	y = 0.001249x + 0.003515	0.9955
CBGA	0.12	0.04	y = 0.000735x + 0.001066	0.9968
CBN	0.85	0.26	y = 0.001434x + 0.003725	0.9928
Δ^9^-THC	0.83	0.25	y = 0.000944x + 0.001867	0.9942
CBNA	0.06	0.02	y = 0.001469x + 0.008459	0.9925
Δ^8^-THC	1.13	0.34	y = 0.000923x + 0.001308	0.9893
CBL	0.38	0.11	y = 0.004237x + 0.007340	0.9895
CBC	1.52	0.46	y = 0.004456x + 0.011457	0.9956
Δ^9^-THCA	0.07	0.02	y = 0.002077x + 0.010347	0.9866
CBT	0.30	0.09	y = 0.006389x + 0.002636	0.9865
CBCA	0.86	0.26	y = 0.000825x + 0.002588	0.9942
CBLA	1.57	0.47	y = 0.000630x + 0.005120	0.9964

**Table 3:** Quantitative analysis of the hemp PT flower sample and comparison with other labs.

Cannabinoids	Mean (w%) (Left) and RSD% (Right) *n* = 3												Bias (%)
	Lab 102		Lab 122		Lab 149		Lab 164		Interlab		Our lab		
CBD	^†^ N/R	N/R	5.3	1.4	3.9	12.6	5.7	4.4	5.0	19.0	5.3	6.2	6.7
CBDA	N/R	N/R	4.8	0.1	3.7	12.8	7.1	3.3	5.2	33.4	4.1	4.1	−21.2
CBC	N/R	N/R	N/R	N/R	N/R	N/R	N/R	N/R	N/R	N/R	0.22	5.7	^‡^ N/A
Δ^9^-THC	0.24	1.3	0.25	1.5	0.22	7.7	0.28	5.1	0.25	10.1	0.19	6.0	−23.2
CBGA	N/R	N/R	N/R	N/R	N/R	N/R	N/R	N/R	N/R	N/R	0.15	5.1	N/A
CBG	N/R	N/R	N/R	N/R	N/R	N/R	N/R	N/R	N/R	N/R	0.14	6.1	N/A
Δ^9^-THCA	0.066	4.9	0.073	0.2	0.081	26.9	0.076	2.9	0.074	14.9	0.043	8.2	−41.9
CBDV	N/R	N/R	N/R	N/R	N/R	N/R	N/R	N/R	N/R	N/R	0.029 *	N/A	N/A
CBN	N/R	N/R	0.045	1.3	0.039	26.8	N/R	N/R	0.043	7.5	0.044	6.3	3.1
CBCA	N/R	N/R	N/R	N/R	N/R	N/R	N/R	N/R	N/R	N/R	0.13	4.4	N/A
CBT	N/R	N/R	N/R	N/R	N/R	N/R	N/R	N/R	N/R	N/R	0.057	4.6	N/A
CBDVA	N/R	N/R	N/R	N/R	N/R	N/R	N/R	N/R	N/R	N/R	0.035	5.4	N/A
Total CBD	N/R	N/R	9.5	0.7	7.2	12.2	11.9	3.4	9.5	24.7	8.9	^§^ N/C	−6.6
Total Δ^9^-THC	0.30	2.0	0.32	1.2	0.29	8.3	0.35	4.5	0.32	8.4	0.23	N/C	−27.0

^†^ N/R, not reported. ^‡^ N/A, not applicable. ^§^ N/C, not calculated. * This value is considered semi-quantitative, as it is slightly below the LOQ (0.032% *w*/*w*) but remains within ±20% of that limit.

**Table 4:** Measured mean (w%) and RSD (%) (*n* = 3) for cannabinoids in six commercial hemp flower samples using the validated LC–MS/MS method.

Cannabinoid	Mean (W%) (Left) and RSD% (Right) *n* = 3											
	CBS		CCS		FVS		LCS		SJS		WCS	
CBDV	^†^ N/D	^‡^ N/A	0.073	3.6	0.23	6.3	N/D	N/A	0.12	5.4	N/D	N/A
CBDVA	0.078	9.9	1.5	3.7	5.3	6.7	N/D	N/A	2.8	9.0	N/D	N/A
CBD	1.7	8.5	0.64	4.0	0.37	4.7	N/D	N/A	0.57	7.9	N/D	N/A
THCV	N/D	N/A	N/D	N/A	0.033	1.4	N/D	N/A	N/D	N/A	N/D	N/A
CBDA	21.1 *	10.1	15.8	6.3	7.6	10.5	N/D	N/A	13.5	9.4	N/D	N/A
CBG	0.12	11.7	0.089	5.0	0.078	11.4	0.39	6.0	0.074	15.9	0.24	5.1
Δ^9^-THC	0.17	10.6	0.064	1.2	0.037	11.4	0.058	5.5	0.055	8.5	0.040	8.9
CBC	0.13	7.8	N/D	N/A	N/D	N/A	0.10	6.9	N/D	N/A	0.12	3.9
CBCA	0.88	9.0	0.64	1.9	0.48	7.8	0.23	5.3	0.57	9.1	0.24	2.3
CBGA	0.38	7.9	0.60	5.0	0.47	5.4	8.2	4.8	0.63	5.4	7.9	6.6
Δ^9^-THCA	0.65	7.4	0.50	4.0	0.28	7.3	0.053	4.5	0.46	8.2	0.043	7.9
THCVA	N/D	N/A	0.070	0.7	0.21	8.0	N/D	N/A	0.12	9.3	N/D	N/A
Total CBD	20.2	N/A	14.5	N/A	7.1	N/A	0	N/A	12.4	N/A	0	N/A
TotalΔ^9^-THC	0.74	N/A	0.50	N/A	0.29	N/A	0.10	N/A	0.46	N/A	0.078	N/A

^†^ N/D, not detected. ^‡^ N/A, not applicable. * This value is considered semi-quantitative, as it is slightly above the upper limit of quantification (ULOQ) (20%, *w*/*w*) but remains within ±15% of that limit.

## Data Availability

The datasets generated during and/or analyzed during the current study are available from the corresponding authors upon reasonable request.

## Supplementary Materials

The following supporting information can be downloaded at: https://www.mdpi.com/article/10.3390/molecules31101684/s1, Figure S1: Chemical structure of the nine isotopically labeled cannabinoids used in this study; Figure S2: LC-MS/MS chromatogram of neutral C21 cannabinoids sharing identical precursor ion (*m*/*z* 315.20). Resolution values between adjacent peaks are 15.53, 1.89, 5.83, 2.58, and 2.77, respectively, for CBD/Δ^9^-THC, Δ^9^-THC/ Δ^8^-THC, Δ^8^-THC/CBL, CBL/CBC, and CBC/CBT; Figure S3: LC-MS/MS chromatogram of acidic C21 cannabinoids sharing identical precursor ion (*m*/*z* 359.20 and 357.20 for positive and negative mode, respectively). Resolution values between adjacent peaks are 22.32, 4.73, and 3.10, respectively, for CBDA/ Δ^9^-THCA, Δ^9^-THCA/CBCA, and CBCA/CBLA; Figure S4: LC-MS/MS chromatogram of neutral varin cannabinoids sharing identical precursor ion (*m*/*z* 287.20), including CBDV and THCV. Resolution value is 9.85; Figure S5: LC-MS/MS chromatogram of acidic varin cannabinoids sharing identical precursor ion (*m*/*z* 331.20 and 329.20 for positive and negative mode, respectively), including CBDVA and THCVA. Resolution value is 14.20; Figure S6: A representative LC-MS/MS chromatogram of the CBS hemp flower extract at 25,000 ng/mL; Figure S7: A representative LC-MS/MS chromatogram of the CCS hemp flower extract at 25,000 ng/mL; Figure S8: A representative LC-MS/MS chromatogram of the FVS hemp flower extract at 25,000 ng/mL; Figure S9: A representative LC-MS/MS chromatogram of the LCS hemp flower extract at 25,000 ng/mL; Figure S10: A representative LC-MS/MS chromatogram of the SJS hemp flower extract at 25,000 ng/mL; Figure S11: A representative LC-MS/MS chromatogram of the WCS hemp flower extract at 25,000 ng/mL; Table S1: Linearity (R^2^) for all analytes. The highest and lowest values were identified and highlighted in red; Table S2: Accuracy of the QC samples: average accuracy values (expressed as percent of nominal concentration) were determined from triplicate measurements under both intraday and interday conditions. The highest and lowest recovery values at each QC level under both intraday and interday conditions were identified and highlighted in red; Table S3: Precision of the QC samples: average precisions were determined from triplicate measurements under both intraday and interday conditions. The highest RSD at each QC level under both intraday and interday was identified and highlighted in red; Table S4: Uncertainty estimates for cannabinoid quantification.

## References

[1] Avoseh F.T., Mtunzi F.M., Avoseh O.N., Takaidza S. (2025). *Cannabis sativa*; Ethnobotanicals, Classifications, Pharmacology, and Phytochemistry. Nat. Prod. Commun..

[2] Radwan M.M., Chandra S., Gul S., Elsohly M.A. (2021). Cannabinoids, Phenolics, Terpenes and Alkaloids of *Cannabis*. Molecules.

[3] Abu-Sawwa R., Scutt B., Park Y. (2020). Emerging Use of Epidiolex (Cannabidiol) in Epilepsy. J. Pediatr. Pharmacol. Ther..

[4] Falcone Ferreyra M.L., Rius S.P., Casati P. (2012). Flavonoids: Biosynthesis, biological functions, and biotechnological applications. Front. Plant Sci..

[5] Tanney C.A.S., Backer R., Geitmann A., Smith D.L. (2021). *Cannabis* Glandular Trichomes: A Cellular Metabolite Factory. Front. Plant Sci..

[6] Duchateau C., Stévigny C., Waeytens J., Deconinck E. (2025). Chromatographic and Spectroscopic Analyses of Cannabinoids: A Narrative Review Focused on *Cannabis* Herbs and Oily Products. Molecules.

[7] Dussy F.E., Hamberg C., Luginbühl M., Schwerzmann T., Briellmann T.A. (2005). Isolation of Δ9-THCA-A from hemp and analytical aspects concerning the determination of Δ9-THC in cannabis products. Forensic Sci. Int..

[8] Johnson J.V., Christensen A., Morgan D., Basso K.B. (2020). Gas chromatography/electron ionization mass spectrometry (GC/EI-MS) for the characterization of phytocannabinoids in *Cannabis sativa*. Compr. Anal. Chem..

[9] De Prato L., Timmins M., Ansari O., Ruthrof K.X., Hardy G.E.S.J., Howieson J., O’Hara G. (2022). Semi-quantitative analysis of cannabinoids in hemp (*Cannabis sativa* L.) using gas chromatography coupled to mass spectrometry. J. Cannabis Res..

[10] Micalizzi G., Cucinotta L., Chiaia V., Alibrando F., Cannizzaro F., Branca G., Maida P., Oliveri P., Mondello L., Sciarrone D. (2024). Profiling of seized *Cannabis sativa* L. flowering tops by means of microwave-assisted hydro distillation and gas chromatography analyses. J. Chromatogr. A.

[11] Delgado-Povedano M.M., Sánchez-Carnerero Callado C., Priego-Capote F., Ferreiro-Vera C. (2020). Untargeted characterization of extracts from *Cannabis sativa* L. cultivars by gas and liquid chromatography coupled to mass spectrometry in high resolution mode. Talanta.

[12] Song L., Provis J., Al-Bataineh A.M., Fabien K.J., Kotler M. (2024). Development of a liquid chromatographic method with a different selectivity for the quantification of eighteen phytocannabinoids in hemp. Talanta Open. Talanta Open.

[13] Galettis P., Williams M., Gordon R., Martin J.H. (2021). A Simple Isocratic HPLC Method for the Quantitation of 17 Cannabinoids. Aust. J. Chem..

[14] Elhendawy M.A., Radwan M.M., Ibrahim E.A., Wanas A.S., Chandra S., Godfrey M., ElSohly M.A. (2024). Validation and Quantitation of Fifteen Cannabinoids in Cannabis and Marketed Products Using High-Performance Liquid Chromatography-Ultraviolet/ Photodiode Array Method. Cannabis Cannabinoid Res..

[15] Zivovinovic S., Alder R., Allenspach M.D., Steuer C. (2018). Determination of cannabinoids in *Cannabis sativa* L. samples for recreational, medical, and forensic purposes by reversed-phase liquid chromatography-ultraviolet detection. J. Anal. Sci. Technol..

[16] Hädener M., König S., Weinmann W. (2019). Quantitative determination of CBD and THC and their acid precursors in confiscated cannabis samples by HPLC-DAD. Forensic Sci. Int..

[17] Correia B., Ahmad S.M., Quintas A. (2023). Determination of phytocannabinoids in cannabis samples by ultrasound-assisted solid-liquid extraction and high-performance liquid chromatography with diode array detector analysis. J. Chromatogr. A.

[18] Meyer G., Adisa M., Dodson Z., Adejumo E., Jovanovich E., Song L. (2024). A liquid chromatography electrospray ionization tandem mass spectrometry method for quantification of up to eighteen cannabinoids in hemp-derived products. J. Pharm. Biomed. Anal..

[19] Woźniczka K., Trojan V., Urbanowicz K., Schreiber P., Zadrożna J., Bączek T., Smoleński R.T., Roszkowska A. (2024). In vivo profiling of phytocannabinoids in *Cannabis* spp. varieties via SPME-LC-MS analysis. Anal. Chim. Acta.

[20] Citti C., Linciano P., Panseri S., Vezzalini F., Forni F., Vandelli M.A., Cannazza G. (2019). Cannabinoid profiling of hemp seed oil by liquid chromatography coupled to high-resolution mass spectrometry. Front. Plant Sci..

[21] McRae G., Melanson J.E. (2020). Quantitative determination and validation of 17 cannabinoids in cannabis and hemp using liquid chromatography-tandem mass spectrometry. Anal. Bioanal. Chem..

[22] Nemeškalová A., Hájková K., Mikulů L., Sýkora D., Kuchař M. (2020). Combination of UV and MS/MS detection for the LC analysis of cannabidiol-rich products. Talanta.

[23] Tran J., Elkins A.C., Spangenberg G.C., Rochfort S.J. (2022). High-Throughput Quantitation of Cannabinoids by Liquid Chromatography Triple-Quadrupole Mass Spectrometry. Molecules.

[24] Song L., LeBlanc L., Jovanovich E., Mohammad Al-Bataineh A., Jervelle Fabien K. (2024). A rapid and accurate liquid chromatographic method for hemp compliance testing. Forensic Chem..

[25] Song L., Valenzuela G., Carlson S., Dodson Z., Adisa M. (2022). Potency testing of up to twenty cannabinoids by liquid chromatography diode array detector with optional electrospray ionization time-of-flight mass spectrometry. Anal. Chim. Acta.

[26] Owolabi A., Ogunsola O., Joens E., Kotler M., Song L. (2025). A Systematic Study of Liquid Chromatography in Search of the Best Separation of Cannabinoids for Potency Testing of Hemp-Based Products. Molecules.

[27] Citti C., Russo F., Sgrò S., Gallo A., Zanotto A., Forni F., Vandelli M.A., Laganà A., Montone C.M., Gigli G. (2020). Pitfalls in the analysis of phytocannabinoids in cannabis inflorescence. Anal. Bioanal. Chem..

[28] (2017). General Requirements for the Competence of Testing and Calibration Laboratories.

[29] Capriotti A.L., Cannazza G., Catani M., Cavaliere C., Cavazzini A., Cerrato A., Citti C., Felletti S., Montone C.M., Piovesana S. (2021). Recent applications of mass spectrometry for the characterization of cannabis and hemp phytocannabinoids: From targeted to untargeted analysis. J. Chromatogr. A.

[30] Palmieri S., Mascini M., Ricci A., Fanti F., Ottaviani C., Lo Sterzo C., Sergi M. (2019). Identification of *Cannabis sativa* L. (hemp) Retailers by Means of Multivariate Analysis of Cannabinoids. Molecules.

[31] Meng Q., Buchanan B., Zuccolo J., Poulin M.M., Gabriele J., Baranowski D.C. (2018). A reliable and validated LC-MS/MS method for the simultaneous quantification of 4 cannabinoids in 40 consumer products. PLoS ONE.

[32] Wilson W.B., Urbas A.A., Abdur-Rahman M., Romares A., Mistek-Morabito E. (2024). Determination of Δ9-THC, THCA, Δ8-THC, and total Δ9-THC in 53 smokable hemp plant products by liquid chromatography and photodiode array detection. Forensic Chem..

[33] Wilson W.B., Abdur-Rahman M. (2022). Determination of 11 Cannabinoids in Hemp Plant and Oils by Liquid Chromatography and Photodiode Array Detection. Chromatographia.

[34] Chen X., Deng H., Heise J.A., Puthoff D.P., Bou-Abboud N., Yu H., Peng J. (2021). Contents of cannabinoids in hemp varieties grown in Maryland. ACS Omega.

[35] Gülck T., Møller B.L. (2020). Phytocannabinoids: Origins and Biosynthesis. Trends Plant Sci..

[36] Hanuš L.O., Meyer S.M., Muñoz E., Taglialatela-Scafati O., Appendino G. (2016). Phytocannabinoids: A unified critical inventory. Nat. Prod. Rep..

[37] ElSohly M.A., Gul W. (2014). Constituents of *Cannabis sativa*. Handbook of Cannabis.

