# Supplementary material for: High-Throughput LC–MS/MS Quantification of Eighteen Cannabinoids in Hemp Flowers with Baseline Separation of Structural Isomers
Source: Molecules. 2026 May 16;31(10):1684. doi: 10.3390/molecules31101684 (PMC13209672; doi:10.3390/molecules31101684)
Supplement: Supplementary file 1 [file molecules-31-01684-s001.zip › molecules-4258090-supplementary.pdf]

High-Throughput LC–MS/MS Quantification of Eighteen Cannabinoids in Hemp Flowers with Baseline Separation of Structural Isomers

Na Liu [1,*], Maggie Schoener [2], Naima Jannath Rimi [2], Md Imon Hossain [2], Supraja Regunathan [3], Robert Powers [3], and Liguo Song [2,*]

[1] Department of Physical and Biological Sciences, Western New England University, Springfield, MA, 01119, USA
[2] Department of Chemistry, Western Illinois University, Macomb, IL 61455, USA
[3] Department of Forensic Science, University of New Haven, West Haven, CT, 06516, USA
[*] Correspondences: NL, na.liu@wne.edu; LS, l-song@wiu.edu

Supplementary Materials

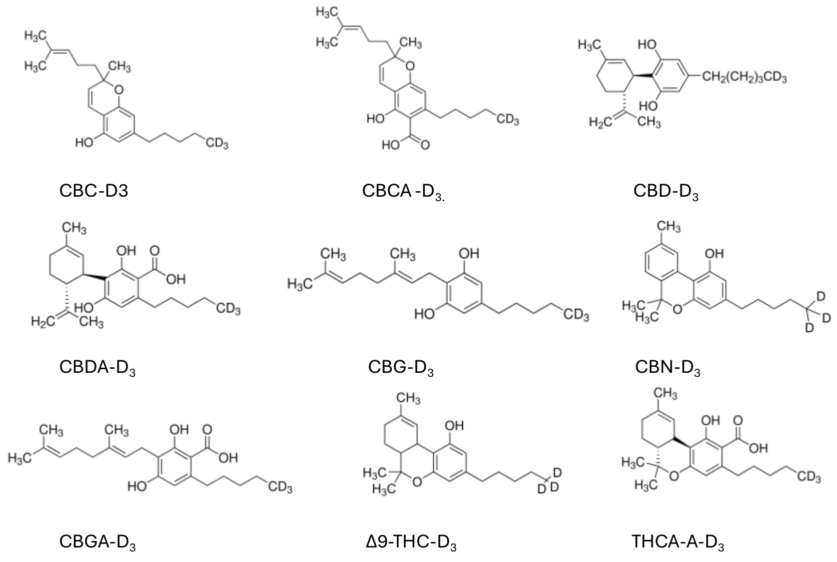

Supplementary Figure S1: Chemical structure of the nine isotopically labelled cannabinoids used in this study.

Supplementary Table S1: Linearity (R^2) for all analytes. The highest and lowest values were identified and highlighted in red.

Linearity (R^2)	CBDV	CBDVA	CBD	THCV	CBDA	CBG	CBN	CBNA	Δ^9-THC	Δ^8-THC	CBL	CBC	CBT	CBCA	CBLA	CBGA	Δ^9-THCA	THCVA
Day 1	0.9983	0.9929	0.9912	0.9937	0.9945	0.9996	0.9928	0.9925	0.9942	0.9893	0.9895	0.9956	0.9865	0.9942	0.9964	0.9968	0.9866	0.9955
Day 2	0.9934	0.9910	0.9842	0.9866	0.9933	0.9904	0.9908	0.9896	0.9920	0.9886	0.9862	0.9903	0.9831	0.9946	0.9957	0.9982	0.9915	0.9950
Day 3	0.9970	0.9913	0.9910	0.9969	0.9932	0.9988	0.9940	0.9916	0.9935	0.9950	0.9940	0.9963	0.9974	0.9908	0.9912	0.9990	0.9904	0.9975

Supplementary Table S2: Accuracy of the QC samples: average accuracy values (expressed as percent of nominal concentration) were determined from triplicate measurements under both intraday and interday conditions. The highest and lowest recovery values at each QC level under both intraday and interday were identified and highlighted in red.

QC levels (ng/mL)	Accuracy (%)	CBDV	CBDVA	CBD	THCV	CBDA	CBG	CBN	CBNA	Δ^9-THC	Δ^8-THC	CBL	CBC	CBT	CBCA	CBLA	CBGA	Δ^9-THCA	THCVA
8	Day 1	98.3	101.5	101.5	103.6	100.1	102.8	106.4	105.5	104.2	93.8	92.9	97.9	105.0	99.9	97.5	98.8	87.6	110.8
	Day 2	115.3	110.0	105.7	116.6	105.4	86.4	99.7	106.5	112.4	109.4	106.7	112.9	111.7	103.0	105.7	101.7	104.9	109.3
	Day 3	107.5	116.8	108.0	101.6	103.7	89.4	102.8	113.9	101.4	108.6	106.1	101.6	108.7	99.7	93.3	107.9	98.7	105.7
	Interday	107.0	111.4	105.1	107.3	103.1	92.9	102.9	108.6	106.0	103.9	101.9	104.1	108.5	100.9	98.8	102.8	97.1	108.6
200	Day 1	99.6	93.7	91.0	97.2	95.3	104.3	106.2	111.3	97.7	91.6	90.4	102.6	93.1	107.3	104.6	106.7	103.7	112.2
	Day 2	96.9	104.1	89.3	93.1	96.1	95.4	105.3	113.1	92.4	89.8	86.8	98.2	86.3	107.1	99.4	105.5	104.9	104.0
	Day 3	101.9	101.9	93.9	99.1	97.6	89.9	108.0	111.6	95.9	94.8	92.7	97.1	94.8	101.8	106.5	106.1	102.7	107.6
	Interday	99.5	99.9	91.4	96.5	96.3	96.6	106.5	112.0	95.3	92.1	90.0	99.3	91.4	105.4	103.5	106.1	103.8	107.9
5000	Day 1	107.3	110.0	113.1	114.2	110.7	104.8	106.2	86.9	113.1	110.9	113.7	114.3	103.4	94.4	99.1	92.6	88.5	89.7
	Day 2	103.3	113.1	114.0	109.2	108.5	90.3	101.8	86.1	105.1	113.8	113.0	107.7	104.3	104.5	103.5	101.8	86.4	95.6
	Day 3	106.7	113.9	110.2	107.6	107.3	89.7	107.4	86.7	108.7	110.0	113.3	105.4	102.7	90.7	101.2	92.3	85.6	92.0
	Interday	105.8	112.3	112.4	110.3	108.8	94.9	105.2	86.6	108.9	111.6	113.3	109.1	103.4	96.5	101.3	95.6	86.8	92.4

Supplementary Table S3: Precision of the QC samples: average precisions were determined from triplicate measurements under both intraday and interday conditions. The highest RSD at each QC level under both intraday and interday was identified and highlighted in red.

QC levels (ng/mL)	Precision (%)	CBDV	CBDVA	CBD	THCV	CBDA	CBG	CBN	CBNA	Δ^9-THC	Δ^8-THC	CBL	CBC	CBT	CBCA	CBLA	CBGA	Δ^9-THCA	THCVA
8	Day 1	1.6	7.3	4.1	4.9	8.5	3.5	4.1	7.2	0.9	3.9	9.0	4.6	3.9	8.4	5.8	2.2	6.4	0.8
	Day 2	2.7	1.3	3.4	2.1	4.1	5.1	7.8	8.7	5.0	6.9	3.4	5.4	2.6	7.4	0.5	2.8	6.6	4.0
	Day 3	2.5	2.7	1.1	2.4	7.2	6.9	3.0	5.3	7.7	5.3	3.4	3.2	3.7	10.9	9.5	1.8	3.0	1.4
	Interday	7.2	5.4	3.8	7.2	6.3	9.3	5.4	7.2	6.5	8.8	8.2	7.6	4.0	8.0	7.7	4.4	9.2	3.0
200	Day 1	1.5	3.5	3.4	2.6	1.4	3.2	1.9	1.7	1.6	1.9	0.5	1.0	2.0	3.5	2.1	0.7	1.4	1.2
	Day 2	1.5	3.0	1.1	1.8	1.6	1.1	3.8	3.8	1.2	0.9	2.4	2.9	1.4	1.5	3.2	0.9	1.7	0.2
	Day 3	3.2	2.0	3.0	1.6	3.5	2.0	2.6	5.2	2.7	1.1	0.6	2.2	2.2	2.7	0.4	0.7	0.6	0.9
	Interday	2.9	5.4	3.2	3.2	2.3	6.8	2.7	3.4	3.0	2.6	3.1	3.2	4.6	3.5	3.6	0.8	1.5	3.4
5000	Day 1	2.0	1.3	0.3	0.8	1.3	3.2	7.0	1.4	1.3	1.6	1.3	0.9	2.8	1.4	1.3	0.3	5.5	0.7
	Day 2	3.6	0.8	1.3	2.6	4.6	2.4	5.0	1.1	0.8	2.6	0.4	1.7	4.4	0.9	1.6	0.6	0.4	0.6
	Day 3	1.1	0.3	1.9	1.0	4.2	5.9	3.3	0.4	1.5	1.2	4.2	4.3	3.6	2.1	0.3	0.3	0.7	0.7
	Interday	2.8	1.8	1.9	3.0	3.4	8.5	5.2	1.0	3.4	2.3	2.3	4.3	3.3	6.5	2.2	4.9	3.2	2.9

Supplementary Table S4. Uncertainty estimates for cannabinoid quantification.

Analytes	CBDV	CBDVA	CBD	THCV	CBDA	CBG	CBN	CBNA	Δ^9-THC	Δ^8-THC	CBL	CBC	CBT	CBCA	CBLA	CBGA	Δ^9-THCA	THCVA
Uncertainty (%)	8.6	8.4	5.9	8.9	8.0	16.4	8.9	7.7	8.6	9.1	9.1	10.1	7.9	12.0	9.0	6.7	9.3	6.2

Note: Expanded uncertainty values (U) were calculated for each analyte using intraday and interday precision data from QC samples, as reported in Supplementary Table S3. The combined control uncertainty (μ_c) was obtained by pooling the interday relative standard deviations at all control levels using the formula:

$$\mathbf{U}_{\textbf{combined control}} = \left(\sum_{i=1}^{n} \boldsymbol{RSDi} \times \boldsymbol{Sample\ size}\right) / \boldsymbol{Total\ sample\ size}$$

Expanded uncertainty was then calculated as:

$$\mathbf{U}_e = K \times \mathbf{U}_{combined\ control}$$

with a coverage factor k=2, corresponding to an approximate 95% confidence interval. These values provide an estimate of method variability due to repeatability and intermediate precision, in line with ISO 17025 guidelines.

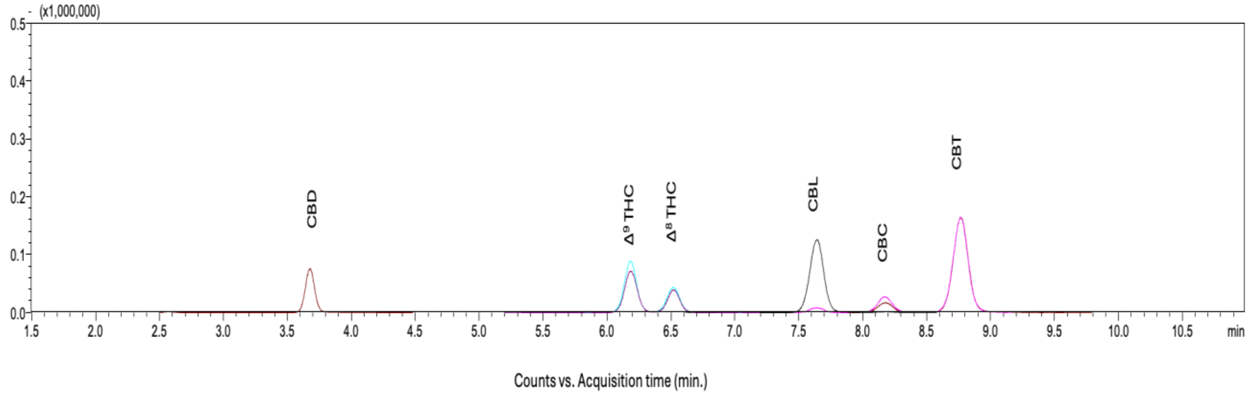

Supplementary Figure S2: LC-MS/MS chromatogram of neutral C21 cannabinoids sharing identical precursor ion (m/z 315.20). Resolution values between adjacent peaks are 15.53, 1.89, 5.83, 2.58, and 2.77, respectively for CBD/Δ⁹-THC, Δ⁹-THC/ Δ⁸-THC, Δ⁸-THC/CBL, CBL/CBC, and CBC/CBT.

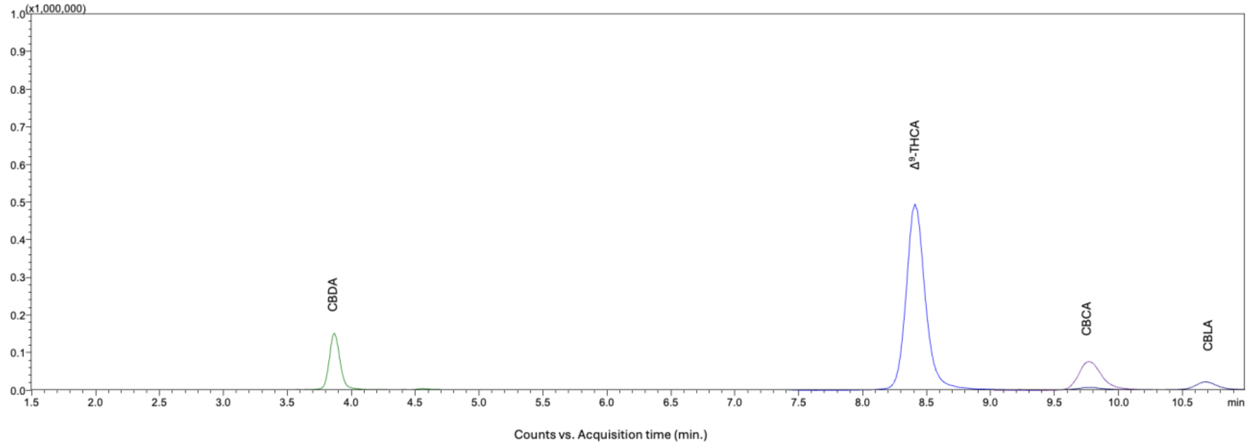

Supplementary Figure S3: LC-MS/MS chromatogram of acidic C21 cannabinoids sharing identical precursor ion (m/z 359.20 and 357.20 for positive and negative mode, respectively). Resolution values between adjacent peaks are 22.32, 4.73, and 3.10, respectively for CBDA/ Δ⁹-THCA, Δ⁹-THCA/CBCA, and CBCA/CBLA.

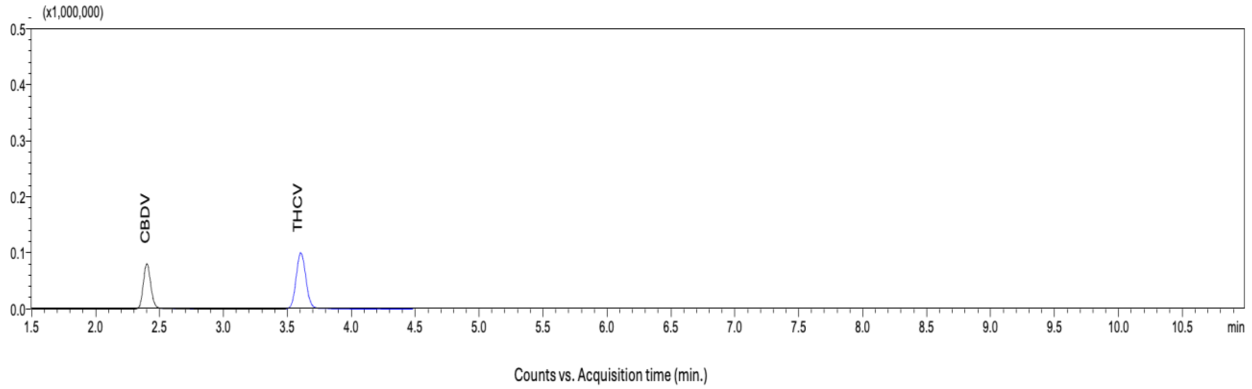

Supplementary Figure S4: LC-MS/MS chromatogram of neutral varin cannabinoids sharing identical precursor ion (m/z 287.20), including CBDV and THCV. Resolution value is 9.85.

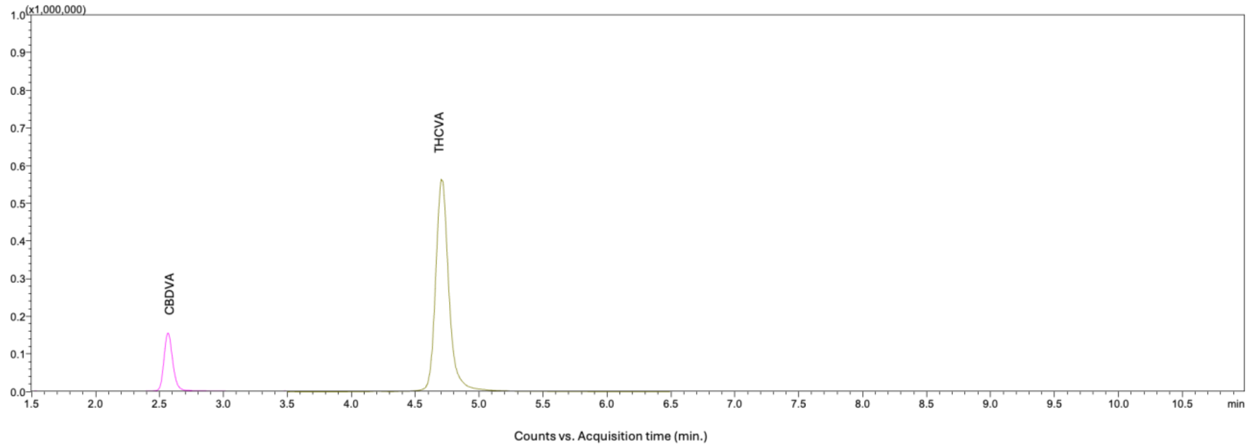

Supplementary Figure S5: LC-MS/MS chromatogram of acidic varin cannabinoids sharing identical precursor ion (m/z 331.20 and 329.20 for positive and negative mode, respectively), including CBDVA and THCVA. Resolution value is 14.20.

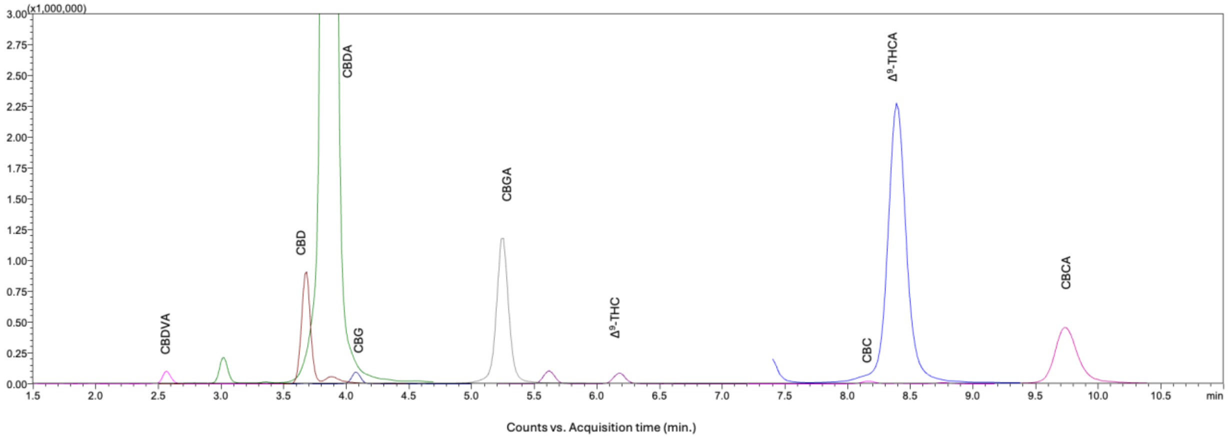

Supplementary Figure S6: A representative LC-MS/MS chromatogram of the CBS hemp flower extract at 25,000 ng/mL

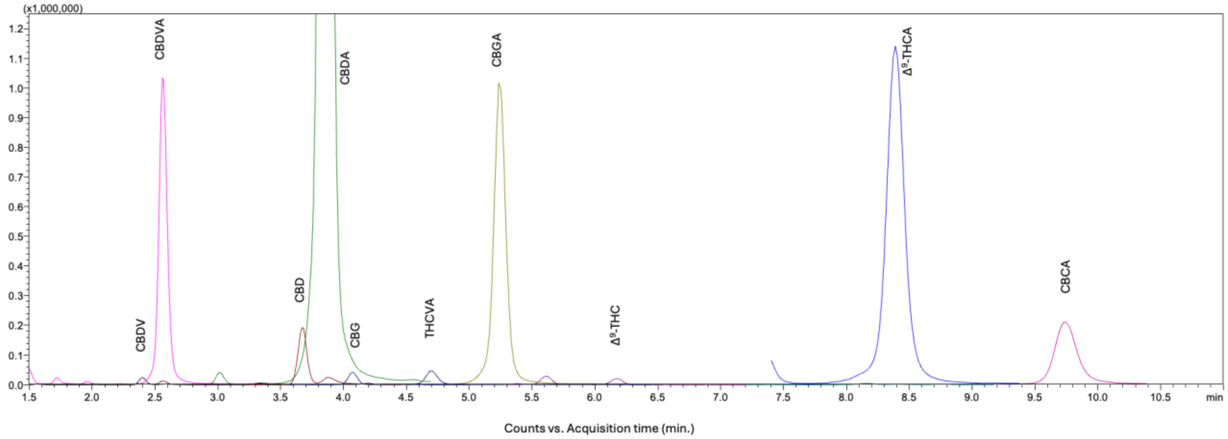

Supplementary Figure S7: A representative LC-MS/MS chromatogram of the CCS hemp flower extract at 25,000 ng/mL

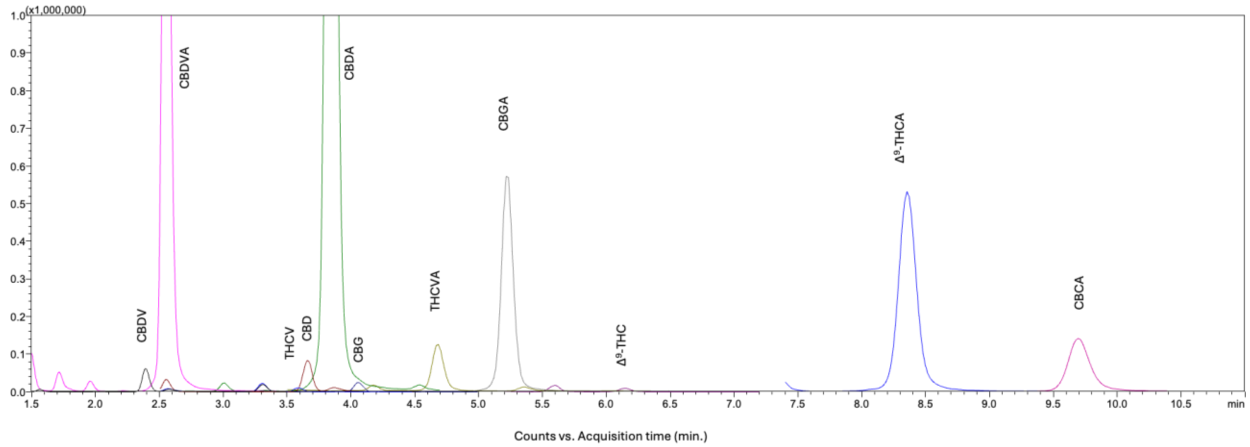

Supplementary Figure S8: A representative LC-MS/MS chromatogram of the FVS hemp flower extract at 25,000 ng/mL

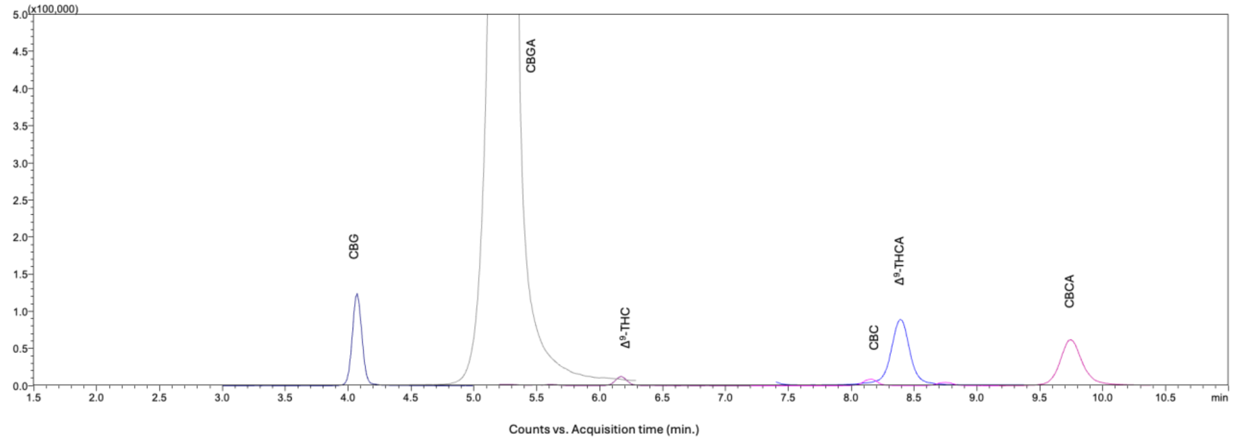

Supplementary Figure S9: A representative LC-MS/MS chromatogram of the LCS hemp flower extract at 25,000 ng/mL

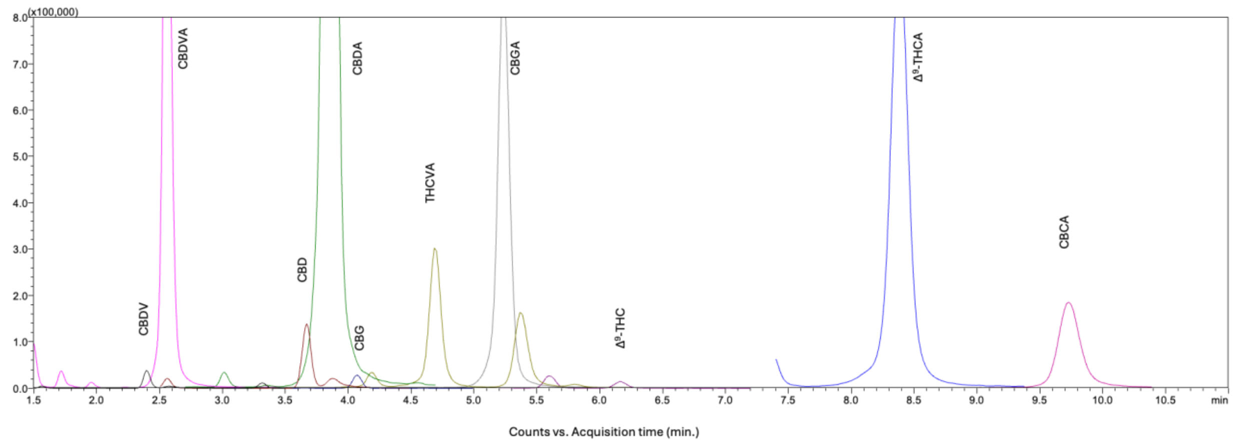

Supplementary Figure S10: A representative LC-MS/MS chromatogram of the SJS hemp flower extract at 25,000 ng/mL

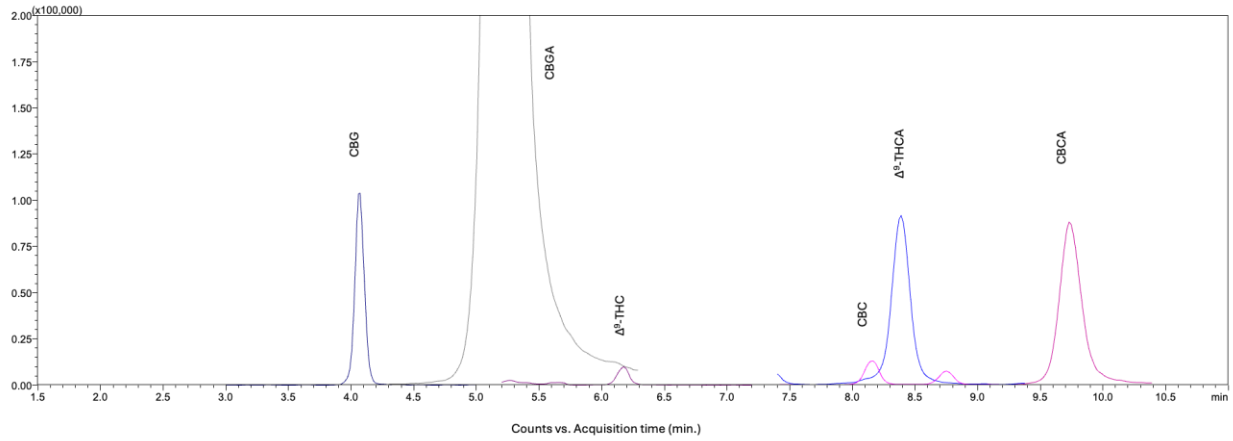

Supplementary Figure S11: A representative LC-MS/MS chromatogram of the WCS hemp flower extract at 25,000 ng/mL
